# The Association of Health Literacy with High-Quality Home Blood Pressure Monitoring for Hypertensive Patients in Outpatient Settings

**DOI:** 10.1155/2020/7502468

**Published:** 2020-03-31

**Authors:** Sau Nga Fu, Man Chi Dao, Carlos King Ho Wong, Bernard Man Yung Cheung

**Affiliations:** ^1^Ha Kwai Chung General Outpatient Clinic, Department of Family Medicine and Primary Health Care, Kowloon West Cluster, Hospital Authority, 77 Lai Cho Road, Kwai Chung, N.T., Hong Kong; ^2^Department of Family Medicine and Primary Care, The University of Hong Kong, 3/F., 161 Main Street, Ap Lei Chau Clinic, Ap Lei Chau, Hong Kong; ^3^Department of Medicine, The University of Hong Kong, Room 405B, 4/F, Professorial Block, Queen Mary Hospital, 102 Pok Fu Lam Road, Pok Fu Lam, Hong Kong

## Abstract

Worldwide hypertension (HT) guidelines recommend use of home blood pressure monitoring (HBPM) in patients with persistent suboptimal blood pressure (BP) readings. It is not clear how patients with limited health literacy could perform HBPM to assist BP control. This study aimed at finding the association between HBPM and patients from lower socioeconomic classes, particularly on the effect of health literacy or educational level. Three electronic databases (MEDLINE, EMBASE, and PubMed) were searched for primary studies with keywords including educational level, health literacy, numeracy, home blood pressure monitoring, accuracy, and quality. The PRISMA guideline was followed. The quality of the literature was assessed by the Cochrane tool and modified Newcastle-Ottawa Scale. Nineteen interventional studies and 29 cross-sectional studies were included. Different populations used different cutoffs to report patients' educational level, whereas health literacy was rarely measured. Three studies used psychometric validated tools to assess health literacy. The quality of HBPM could be assessed by the completion of the procedures' checklist or the number of HBPM readings recorded. The association between subjects' health literacy or educational level and the quality of HBPM was variable. The interventional studies showed that increasing professional-patient contact time could improve patients' knowledge, efficacy, and quality of HBPM. *Conclusion*. Patients' educational level and literacy were not the limiting factors to acquire high-quality HBPM. High-quality HBPM could be achieved by the structured educational intervention. The quality and amount of evidence on this topic are limited. Therefore, further studies are warranted.

## 1. Background

Among hypertensive patients, 10% to 50% of their office blood pressure (BP) readings are higher than the home blood pressure readings [1]. In patients presented with uncontrolled hypertension in our daily practice, home blood pressure monitoring (HBPM) (also known as self-blood pressure monitoring (SBPM)) is an essential monitoring option especially for patients with a suspected white coat effect or masked hypertension. It has become an important recommendation in most international hypertension management guidelines [2–4]. HBPM was also shown to improve office BP readings, increase BP control rate, and enhance the quality of life at low patient risk [5]. The beneficial effect could be reassured when HBPM is delivered with other forms of interventions, such as patient education or drug titration [5, 6]. Therefore, health care professionals routinely recommend HBPM to patients with high office BP.

Compared with standard ambulatory BP measurement, HBPM had mean sensitivity of 85.7% (78.0% to 91.0%) and specificity of 62.4% (48.0% to 75.0%) in diagnosing hypertension [7]. The relatively large range of sensitivity and specificity highlighted various factors affecting the accuracy of HBPM. The HBPM readings may be inaccurate due to patients' inappropriate operation, withholding undesirable readings, or inaccurate automated devices. Parker et al. indicated that there was an end-digit preference for zero numbers and specific-value preference for readings just below the alert threshold among patients in self-reporting their BP [8]. In addition, as there were large varieties of home blood pressure monitors available in the market, their accuracy could be questionable. An assessment done by Ringrose et al. revealed that most home BP devices were not accurate to within 5 mmHg [9].

As a result, health care professionals could prescribe inappropriate management according to inaccurate HBPM records.

From a patient's perspective, performing HBPM is not always an easy task. Patients with lower self-efficacy, lower educational levels, or lower health literacy may need special interventions to assist home monitoring. Fletcher et al. illustrated patients' and health care professionals' concerns of HBPM in a qualitative review, as HBPM involved interpretation, attribution, and action [10]. The quality of HBPM could be highly operator-dependent. Any inaccurate readings or wrong interpretation may lead to patient anxiety, overdiagnosis, or overtreatment due to falsely high home BP readings. On the other hand, falsely low home BP may lead to false reassurance, underdiagnosis, or poor drug compliance.

Different elements are required to perform high-quality HBPM as described in clinical guidelines. They include access to accurate BP monitors, skills, and knowledge to perform HBPM, motivation to perform HBPM regularly, and accurate reflection of HBPM readings to their health care providers. Patients may not have the hardware, skill, and knowledge to implement successful HBPM. They need health care providers' instruction and feedback to practice HBPM independently. Their skills and BP records should be reviewed regularly in order to ensure their compliance with HBPM protocol, such as measurement preparation, procedure, and how to record BP readings. In a busy primary care practice, time constraints may preclude physicians from taking time to educate HBPM and review patients' home BP records.

Conventionally, many studies assessed patients' educational level as part of the sociodemographic background instead of assessing patients' health literacy specifically. “Health Literacy” (HL) is the patient's ability to read, interpret, and respond to the information during health care activities. It was defined by the American Medical Association in 1999 as “the constellation of skills, including the ability to perform basic reading and numeral tasks required to function in the healthcare environment” [11]. Underprivileged patients, such as those from lower socioeconomic class, those with lower educational levels, or those with limited health literacy or numeracy, were found to have a poorer outcome in overall noncommunicable diseases [12]. In addition, patients with inadequate health literacy were more likely to have poorer disease knowledge, poorer self-efficacy, and misconception in cardiovascular disease [13]. They may also encounter greater barriers in performing accurate HBPM. Few studies have focused on whether the underprivileged patients were able to perform HBPM as good as middle or above socioeconomic class patients.

Given the large and increasing global disparities of BP control in hypertensive patients from the low-income population, there is a clinical urge to formulate suitable interventions which could help patients achieve desirable BP targets [14]. Most of the existing review papers focused on the BP outcomes of the global hypertensive population after different HBPM interventions [5–7, 10]. So far, there is a limited understanding of how the socially disadvantaged population could successfully perform high-quality HBPM that could subsequently improve their BP control. This study aimed to find out the association between patient health literacy (including educational level and other related socioeconomic factors) and HBPM, which may or may not lead to improvement of BP control. The finding will be particularly useful to the low-income hypertensive populations.

## 2. Methods

We performed a systematic review using the Preferred Reporting Items for Systematic Reviews and Meta-Analyses (PRISMA) reporting guideline to investigate the association between HBPM and patients' health literacy or educational level [15].

### 2.1. Selection of Studies

We included all original research articles evaluating adult practice or attitude towards HBPM or SBPM, which include knowledge, skills, and practice towards HBPM or SBPM. The articles should contain an association of subjects' ability to read, understand, and follow instructions, such as educational level, health literacy, and numeracy with either HBPM or overall BP control. The articles could include processes of HBPM or SBPM, practices such as the prevalence of HBPM or SBPM, and skills or knowledge of HBPM or SBPM. There was a preferable analysis of the association between study subjects' ability and quality of HBPM or SBPM.

We excluded studies with neither analysis of HBPM or SBPM practice nor subjects' educational level or health literacy.

### 2.2. Search Strategy

We performed a web-based search of the MEDLINE, EMBASE, and PubMed databases. We also screened the reference list of all relevant studies (snowball search). Studies published in English from 1910 to present were included.

We defined two main search concepts (“self-blood pressure monitoring” and “appropriateness of self-care activities”) and combined the search by “AND.” We used the MeSH term “blood pressure monitoring, ambulatory” or the term “self-blood pressure monitoring” or “self-blood pressure measurement,” “home blood pressure monitoring,” “self-measurement,” and “blood pressure.” We then used the term “health literacy,” “mathematics,” “numeracy,” “educational level,” “educational status” or “health knowledge, attitude, practice.” We limited searching by studies for adults (age > 18) The whole syntax is shown in Table 1. The date of the last search was on 27 November 2019.

### 2.3. Selection of Publications

We went through a two-step selection process. We first read the titles and abstracts. Studies meeting all inclusion criteria above were identified as potentially appropriate. We then analyzed the full texts of the selected articles according to the inclusion and exclusion criteria. Reasons for exclusion were documented.

**Figure 1:**
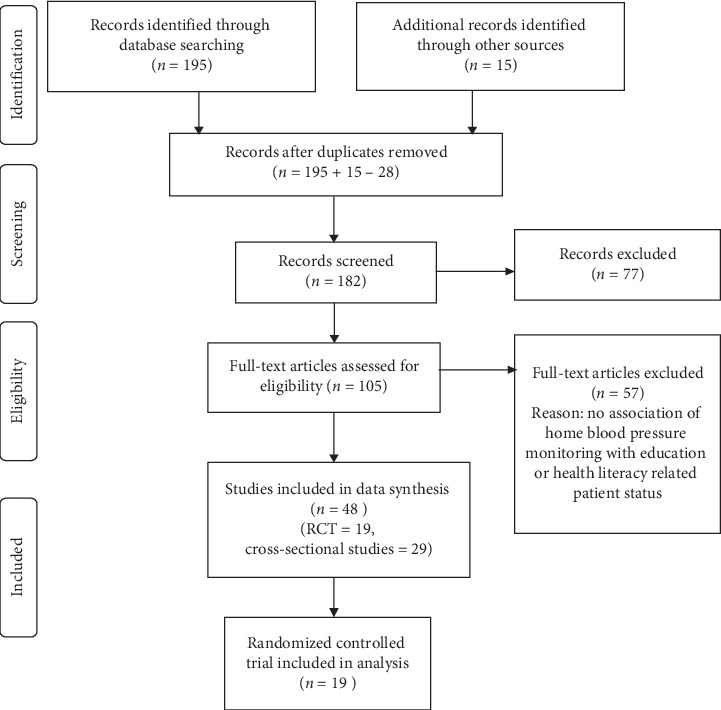
PRISMA flow diagram.

Two independent review authors (SF and MD) did the whole selection process. Disagreements between us were resolved by consensus. A review author (either CW or BC) was consulted if disagreement persisted. The PRISMA flowchart is shown in Figure 1 [16].

The selection of the studies is based on the following criteria:

1. The article is an original study, which includes a detailed study method for the assessment of the risk of bias.
2. The quantitative studies included assess the association between health literacy or numeracy or educational status of patients and their practice or knowledge on SBPM or HBPM or evaluate the interventions to enhance HBPM practice by enhancing the efficacy of HT patients.
3. The patient outcomes of studies involved HBPM attitude, knowledge, and practice; or the outcome involved hypertension BP control.

The studies centered on adult patients with an established diagnosis of hypertension. The studies that focused on diagnostic tests, screening of hypertension, and hypertension in pregnancy were excluded.

### 2.4. Data Extraction

We extracted bibliographic data (author, publication year, title, and journal), study design, setting, country, inclusion and exclusion criteria, subject recruitment, study population characteristic (age and gender), and date and duration of the study. We registered the tools used for assessing the outcome measurement and if there is an association between the subjects' health literacy and the appropriateness of HBPM. We retrieved all outcome categories. Finally, we extracted the HBPM-related interventions and patient outcome particularly for subjects with relatively low health literacy or educational level.

### 2.5. Quality Assessment

Critical appraisal was independently recorded by reviewers to allow comparison. Risk of bias was assessed by considering relevant domains to interventional studies, including participant selection, measurements of variables, and controlling for confounding, in line with the Cochrane Collaboration's Grading of Recommendations Assessment, Development, and Evaluation (GRADE) tool for assessing the risk of bias [17, 18]. Each domain was rated with “high,” “low,” or “unclear” according to the risk of bias, with free text explanations. In order to maximize relevance to nonrandomized studies, the Newcastle-Ottawa Scale (NOS) for cross-sectional studies was used [19]. Two authors (SF and MD) assessed the individual study by three domains which are selection (maximum 5 stars), comparability (maximum 2 stars), and outcome (maximum 3 stars) resulting in total NOS grade. The summation of the 3 domains' number of stars resulted in the total NOS score. Very good studies scored 9-10 stars, good studies scored 7-8 stars, satisfactory studies scored 5-6 stars, and unsatisfactory studies scored 0–4 stars.

### 2.6. Data Synthesis

We separately collected the cross-sectional studies and the interventional studies data for narrative synthesis. Different assessments of educational level and/or health literacy, HBPM device, technique, and quality of HBPM were recorded.

## 3. Results

Figure 1 shows the systematic search and selection of relevant studies adopting the PRISMA guideline 2009 [16]. 195 studies were identified from MEDLINE, EMBASE, and PubMed. Bibliographies of primary studies and review articles meeting the inclusion criteria were searched manually to identify 15 further eligible studies. 182 unique studies in total were included for the screening of abstracts. After reviewing the abstracts, 77 studies were excluded because the studies did not assess HBPM or self-BP monitoring, or the research subjects were not hypertensive patients, nor was there any association between HBPM and patients' educational status or health literacy. 105 studies were included for full-text assessment of eligibility. Finally, 48 studies (19 randomized controlled trials and 29 cross-sectional studies) were included in the data synthesis.

The results of cross-sectional studies are shown in Table 2. The included studies were performed in North America, Europe, and Asia from 2003 to 2019. Most of the participants were patients with hypertension. Two of the studies surveyed pharmacists and primary care providers such as nurses and physicians about their clinical practice of HBPM. Study sites included a populational survey, recruited in community organizations, primary care clinics, or outpatient clinics in the hospital. Most studies demonstrated a positive relationship between subjects' educational level or health literacy is associated with owning BP monitors at home, performing it regularly or recording the measurements accurately. Five out of twenty-nine studies reported a negative association between patient educational level or other social factors and practice of HBPM. The BP outcomes of patients were included in 5 studies: 2 studies showed a positive association of HBPM and BP control, while 3 studies did not demonstrate any better BP control.

The quality assessment by Newcastle-Ottawa Score for cross-sectional studies found the studies ranged from very good (grade 9/10) to unsatisfactory (grade 2/10). Most unsatisfactory studies got low sampling scores.

Table 3 shows the results of the 19 interventional studies. Most studies were performed in North America, while 2 of them were performed in Europe and one of them was performed in Hong Kong. There were different modes of HBPM interventions, such as providing home BP monitors, patient education, and training intervention, record and feedback system to HBPM measurements, training, and updating knowledge to health care providers.

### 3.1. HBPM in the Included Studies

There were various types of home BP monitors involved in selected studies. They included automatic electronic branchial devices, electronic semi-automated branchial devices, manual mercurial sphygmomanometer, and electronic wrist devices. The possession of home BP monitors was related to higher educational level and/or income status [20, 25, 41, 44], while the frequency of HBPM as clinician recommended was not necessarily related to the educational level. Ragot et al. found that 90% of HBPM users did not receive information about HBPM use [37]. Other studies demonstrated that the HBPM quality might not be related to the educational level. When patients were instructed to use HBPM by health care providers, there was consistent reporting of regular HBPM use [22, 28, 36, 46].

There was no psychometrically validated tool to assess the quality of HBPM. The quality of HBPM was assessed by different tools defined by authors in different studies. Some used the number of successfully documented or transmitted BP readings over the number of expected BP readings as high-quality HBPM. Flacco et al., Dymek et al., and Fung et al. used procedure checklists developed according to HBPM guidelines to get the total quality scores [27, 28, 53]. Either a video recording of the HBPM procedure or a direct-observation method could be used to assess the HBPM procedure. Dymek et al. demonstrated a deficiency in both knowledge and skills in HBPM in 14 hypertensive patients, while Flacco et al. showed adequate HBPM quality in more than 80% of the subjects. Merrick et al. assessed HBPM quality by comparing the BP readings by a trained volunteer with that by research subjects [33].

### 3.2. Educational Status

Most studies assessed the subjects' educational status. The educational level was usually self-reported as part of personal characteristics. The assessment method could be highly heterogeneous. Most studies categorized educational attainment into different levels of schools: primary schools, middle schools, high schools, and colleges, but the cutoff level and the number of categories highly varied. Three studies included “illiterate” as one of the educational status categories [25, 43, 45]. Other studies also included years of education for data synthesis. If the number of years was defined as binary categories, their cutoff years could vary from 5 years to 12 years.

### 3.3. Health Literacy

HL was not commonly assessed in studies of HBPM. Only 3 studies used 4 different validated health literacy (or numeracy) scales for assessment. Kim et al. used the High Blood Pressure Health Literacy Scale [57]. They did not categorize the subjects as high or low HL. They measured the change in HL before and after the intervention. Shi et al. used the Chinese Health Literacy Scale for Hypertension [40]. More than half of their study subjects (55.3%) had low health literacy. Rao et al. used the Rapid Estimate of Adult Literacy in Medicine-Short Form (REALM-SF) and the 3-item numeracy measure [38]. Less than one-third of the subjects (31%) had low numeracy. These three studies found a strong association between health literacy or numeracy with educational status.

### 3.4. Other Assessments of Patients' Ability

Apart from educational status and health literacy, six studies quantified the subjects' ability by different knowledge scores. The scoring items included knowledge about hypertension, hypertension complications, hypertension comorbidity, and HBPM [20, 27, 31, 32, 37, 57].

### 3.5. Association between HBPM and Educational Status or Health Literacy

In some studies, subjects with higher educational levels were not found to use more HBPM [21, 28, 29, 31, 35, 64]. However, in one study, a larger proportion of subjects with higher educational levels used HBPM [25]. Subjects who believed HBPM could help BP control performed more regular HBPM [21, 36, 41]. Some studies showed that patients with higher educational levels, higher HL, or higher numeracy could perform higher quality of HBPM, such as better compliance with HBPM procedure and more complete or accurate HBPM record [26, 38, 45, 47]. Flacco et al. did not find such an association. Instead, they found that patients performed higher quality of HBPM if they received HBPM instructions from pharmacists or doctors than if they received them from nonprofessionals [28].

### 3.6. Interventions to Improve HBPM for Patients with Different HLs

The interventional studies described different complex interventions that targeted patients with uncontrolled hypertension. Some of them targeted socially disadvantaged subjects, i.e., Korean American older patients and African Americans [57, 62]. Kim et al. used multiple patient educational sessions to focus on hypertension management skill building, HL training, followed by telephone counselling and HBPM. It is reflected that patients had improvement in HL, self-efficacy, and BP control after the intervention. In another study, Ogedegbe et al. used computerized interactive patient education modules, lifestyle counselling, HBPM, clinicians' continuous medical education training, clinicians' case round, and audit of patients' BP reading. They demonstrated that patients with moderate to good HL had marginal significant improvement in BP control [62]. Most interventions resulted in improvement in BP readings or BP control rate, while some reported no significant difference in BP outcomes. Few studies analyzed the elements that resulted in better BP control. Morgado et al. showed improvement in both patient drug adherence and BP control [60, 61].

## 4. Discussion

In this review, the educational status might or might not be associated with HBPM practice, quality, and compliance. The finding was particularly significant for patients with lower socioeconomic status. It was known that patients with lower educational status and lower income had a higher risk of hypertension and more nonadherence to antihypertensive treatment, subsequently leading to poorer clinical outcomes [67]. Health care providers could consider HBPM as an intervention which could improve drug adherence and BP control. Important elements included coaching patients on proper selection of HBPM devices and correct HBPM techniques (e.g., accurate recording of home BP readings). It could be done by providing home devices that store multiple BP readings, or uploading readings to smartphones or computers, or transmitting them directly to electronic health records.

Although various hypertension guidelines recommended the use of HBPM in diagnosis, the overall possession and compliance to HBPM were suboptimal.

We also identified that after structured training, socially disadvantaged patients could have significant improvement in HL, self-efficacy, and BP control. We suggested a structured intervention for identifying patients with low HL and offering training of hypertension self-care including HBPM, with the effect of improving BP control and increasing patients' HL. In view of the fact that less than 40% of the hypertensive patients had optimal BP control in different populations, the promotion of high-quality HBPM in patients with uncontrolled hypertension should be a clinical priority.

There was no structural or validated tool for assessing the quality of HBPM. The quality of HBPM depends on a validated BP device, competence of patients to perform HBPM on their own with a correct method and frequency, a record of accurate HBPM reading, and sharing of that record with health care professionals. Studies in this review modified recommendations of HBPM procedures from various international guidelines. Some studies also adopted the teletransmission of BP readings via the electronic system. We, therefore, suggest future research to develop a patient-friendly protocol to assess high-quality HBPM.

We also found that only a small proportion of studies focused on the assessment of HL and the outcome of hypertension control. The most common assessment is the educational status, which may be unrelated to patients' performance or compliance with antihypertensive treatment. The EHS Guideline proposed that the first step to tackle patients with poorly controlled chronic illness should be patient-centered care: to identify patients' barriers to better control the disease [1]. For instance, health care professionals should be well-equipped with communication techniques with low HL patients. Rajah et al. summarized that healthcare professionals should use everyday language and teach-back method and provide patients with reading materials and aids. However, the most commonly reported barrier regarding patient-centered care is time constraints.

### 4.1. Strength of the Study

This is the first study focusing on HBPM and its association with patients' education level, including health literacy, numeracy, and other socially related factors. Most studies were performed in community or outpatient settings, where primary care observation or intervention could be applied.

### 4.2. Limitations of the Study

Heterogeneous assessment of HBPM or SBPM, the prevalence of HBPM, and the educational status of patients are limitations of this study. Validated assessment of health literacy is sparse.

## 5. Conclusion

Patients' educational or health literacy levels were not limiting factors to acquire skills and knowledge of HBPM. High-quality HBPM could be achieved by structured educational interventions. Complex interventions involving patient education, providing valid home BP monitors, and facilitating patient-clinician communication may improve BP control. Those interventions should be tailor-made to subjects with low educational levels, which could be equally effective in improving the overall BP control.

## Figures and Tables

**Table 1:** Searching strategies.

PubMed	MEDLINE	EMBASE
(1) (“Health literacy” [MeSH] OR (2) “Patient education” as Topic [MeSH] OR (3) “Health status disparities” [MeSH] OR (4) “Educational status” [MeSH] OR (5) “Health education” [MeSH]) AND (6) Blood pressure monitoring, ambulatory [MeSH major topic] (7) Limited to English language	(1) Home monitoring/or home care/ (2) Reading/or health education/or health literacy/ (3) Patient compliance/ (4) Education program/or interdisciplinary education/or education/or health education/or patient education (5) Blood pressure monitoring, ambulatory (6) Self-care/ (7) Educational status/ (8) Patient education as topic/ (9) “Reproducibility of results”/ (10) Essential hypertension/or White coat hypertension/or masked hypertension/or MEDLINE / (11) 1 or 5 or 6 (12) 2 or 4 or 7 or 8 (13) 3 or 9 (14) 10 and 11 and 12 and 13 (15) Limit 14 to (English language and (MEDLINE or “PubMed not MEDLINE”))	(1) Blood pressure monitoring/ (2) Home monitoring/or home care/ (3) Self-monitoring/ (4) Reading/or health education/or health literacy/5. Health disparity/6. Patient compliance/ (7) Reproducibility/ (8) Essential hypertension/ (9) White coat hypertension/ (10) 1 or 2 or 3 (11) 8 or 9 (12) Education program/or interdisciplinary education/or health education/or patient education/ (13) 4 or 5 or 12 (14) 10 and 11 and 13 (15) Limit 14 to (English language and (EMBASE or MEDLINE))
70 items found	63 items found	62 items found

**Table 2:** Summary of the selected cross-sectional studies.

Lead author [Ref], country, year	*N*	Level of education, health literacy, or other social factors	Any association between educational level and HBPM?	Any association between educational level and better BP control?	NOS grading
Allibe et al. [20], France, 2016	380	Education level <A-level, A-level, >A-level hypertension knowledge	NA	Yes, knowing the correct BP target is significantly associated with normal BP	S^*∗*^C O^*∗*^ 2/10 = unsatisfactory
Ayala et al. [13], USA, 2017	559	≤High school graduate, some college graduate, or more	Yes, older age, and those who believe lower BP can reduce the risk of heart attack and stroke had higher % of HBPM Educational level is not associated with HBPM use	NA	S^*∗∗*^C^*∗∗*^O^*∗∗*^ 6/10 = Satisfactory
Ayala et al. [21], USA, 2008	3739	<High school graduate high School graduate Some college College graduate or more	neutral, regular HBPM users had an insignificantly higher educational level Subjects who perceived HBPM helped control their BP Measured BP more frequently	NA	S^*∗∗∗*^C^*∗∗*^O^*∗∗*^ 7/10 = Good
Bancej et al. [22], Canada, 2010	6142	Educational attainment Less than secondary school Secondary school graduate Some after secondary Postsecondary graduate	Yes, regular HBPM was more likely among older adults; those who believed to control BP; and those who had been shown how to perform HBPM by a health professional No, HBPM practice was not related to the level of education	NA	S^*∗∗∗*^C^*∗∗*^O^*∗∗*^ 7/10 = Good
Breaux-Shropshire et al. [23], USA, 2012	149	NA	NA	No, HBPM was not a predictor of blood pressure control	S^*∗*^*∗∗*C^*∗∗*^O^*∗*^ 6/10 Satisfactory
Cacciolati et al. [24], France, 2012	1,814	High: ≥12 years formal education Low: <12 years of formal education Cognitive level: MMSE autonomy: Lawton scale Five basic daily activities	Yes, less HBPM in subjects age >80, with lower educational level and those had no autonomy	NA	S^*∗∗∗∗*^C^*∗∗*^O^*∗∗*^ 8/10 = Good
Cai et al. [25], China, 2017	1878	Illiterate/primary or above	Yes, those with higher education were more likely to perform HBPM	NA	S^*∗∗*^C^*∗∗*^O^*∗∗∗*^ 7/10 = Good
Cuspidi et al. [26], Italy, 2005	855	Primary/secondary/tertiary year of education	Yes, those with higher educational level used HBPM more frequently	NA	S^*∗∗*^C^*∗∗*^O^*∗∗∗*^ 7/10 = Good
Dymek et al. [27], Poland, 2015	14	10 items patients' knowledge score Primary, secondary, university, or above	Yes, overall subjects had fair compliance to HBPM Results showed deficiency in both knowledge and skills	NA	S^*∗*^C O^*∗∗*^ 3/10 = unsatisfactory
Flacco et al. [28], Italy, 2015	725	None/elementary Middle/high school Bachelor/higher	No, high-quality HBPM is not related to the educational level Yes, better quality if subjects received HBPM instructions from doctors or pharmacists	NA	S^*∗*^C^*∗∗*^O^*∗∗*^ 5/10 Satisfactory
Gohar et al. [29], UK, 2008	153	Mean years in education = 12.25 years	No, it was not associated with gender, alternative or complementary medicine use, or adherence to medication	NA	S^*∗*^CO^*∗*^ 2/10 = unsatisfactory
Hu et al. [30], China, 2013	318	Years of education ≤6 years; >6 years	Yes, older participants (>or = 65) were more likely to perform HBPM No, educational level is not related to the practice of HBPM	NA	S^*∗∗*^C O^*∗∗*^ 4/10 = unsatisfactory
Kim et al. [31], USA, 2010	377	Scoring of high BP knowledge Cut-off at <90th percentile or ≥90th percentile	No, compliance with HBPM is not associated with HT knowledge or educational level	NA	S^*∗∗∗*^C^*∗∗*^O^*∗∗∗*^ 8/10 = Good
Melnikov [32], Israel, 2019	430	Years of education Total hypertension knowledge score	Yes, more years of education and those who performed HBPM had better knowledge of hypertension	NA	S^*∗∗*^C^*∗∗*^O^*∗∗*^ 6/10 = Satisfactory
Merrick et al. [33], USA, 1997	91	Years of education Cutoff <12 years & ≥12 years	No, the accuracy of BP measurement is not related to the factors assessed	NA	S^*∗*^C O^*∗∗*^ 3/10 = unsatisfactory
Milot et al. [34], Canada, 2015	1010 (2010) 1005 (2014)	Received HBPM recommendations from their doctors	Only 15% of patients in 2010 and 18% in 2014 were defined as sufficiently compliant with all HBPM procedures	NA	S^*∗∗*^C^*∗*^O^*∗*^ 4/10 = unsatisfactory
Mitchell et al. [35], USA, 2015	193	College graduate/some college/< high school	No, HBPM is not associated with BP levels, age, sex, race, or education level	NA	S^*∗∗*^C O^*∗*^ 3/10 = unsatisfactory
Naik et al. [36], USA, 2008	212	Older adults, some college education Self-management behaviors Communication factors	Yes, patients' endorsement of a shared decision-making style is associated with more HBPM	Yes, proactive communication with one's clinician about abnormal HBPM is associated with better BP control	S^*∗∗∗∗∗*^C^*∗∗*^O^*∗∗*^ 9/10 = very good
Ragot et al. [37], France, 2005	104 pharmacists 1015 patients	Patients' knowledge for lifestyle change for HT, equipped with an automatic HBPM device, knew the name of drugs, treatment-related side effects, and drug compliance	Yes, 90% reported using the device without any rule. In all, 10% of the patients followed doctor's or pharmacist's recommendations	No, those had higher educational level had better hypertension knowledge, but were not better BP controlled	S^*∗*^C^*∗∗*^O^*∗*^ 4/10 = unsatisfactory
Rao et al. [38], USA, 2015	409	Some high school, high school graduate, some college, college graduate Rapid estimate of adult literacy in medicine-short form (REALM-SF) numeracy: 3-item numeracy measure	Yes, adequate numeracy, but not high literacy is associated with more complete reporting of HBPM	NA	S^*∗∗∗*^C^*∗∗*^O^*∗∗∗*^ 8/10 = Good
Seidlerová et al. [39], Czech, 2014	449	Primary, secondary, university	Yes, older age, university education, married, and longer duration of HT were more likely to have HBPM device Regular HBPM is associated with the no. of HT drugs	No, BP control is not associated with frequency of HBPM	S^*∗∗*^C^*∗∗*^O^*∗∗*^ 6/10 = Satisfactory
Shi et al. [40], China, 2017	523	Primary, middle, high school, higher education Chinese Health Literacy Scale for Hypertension	Yes, higher HL was more compliant with HBPM	NA	S^*∗∗∗*^C^*∗∗*^O^*∗*^ = 6/10 Satisfactory
Tan et al. [41], Singapore, 2005	224	None and primary Secondary Tertiary/poly/graduate	Yes, HBPM use was associated with higher-income status Nonusers were associated with failure to recognize benefits, HBPM awareness, understanding of device operation, and perception of HBPM inaccuracy	NA	S^*∗∗∗*^C^*∗∗*^O^*∗∗*^ 7/10 = Good
Tirabassi et al. [42], USA, 2013	1254	Different primary care providers (PCPs)	Yes, PCPs were less likely to recommend HBPM to their patients if they were from poor to the lower middle class than those PCPs with most patients from higher economic classes	NA	S^*∗∗∗*^C^*∗∗*^O^*∗∗*^ 7/10 = Good
Tekin et al. [43], Turkey, 2012	2747	Illiterate Literate/primary school Graduate Middle school graduate High school graduate University graduate	Yes, higher educational level and higher-income level are associated with possession of HBPM	NA	S^*∗∗∗*^C^*∗∗*^O^*∗∗∗*^ 8/10 = Good
Tyson and Mcelduff [44], UK, 2003	222	College or university	Yes, subjects who had further education were more likely to own HBPM and participate in monitoring	NA	S^*∗∗*^C^*∗∗*^O^*∗∗*^ 6/10 = Satisfactory
Uzun et al. [45], Turkey, 2009	150	Illiterate Literate but no graduation Graduated from elementary school Junior high school High school License program	Yes, informed about HT & CVD risk factors informed is better and education level (higher is better)	NA	S^*∗*^C^*∗∗*^O^*∗∗∗*^ 6/10 Satisfactory
Viera et al. [46], USA, 2008	530	<High school graduate High school graduate Some college or more	Yes, 35.2% of patients report that their physicians had recommended HBPM to them	NA	S^*∗∗∗∗∗*^C^*∗∗*^O^*∗∗*^ 9/10 = very good
Wang et al. [47], China, 2014	1915	Junior high school Senior high school College	Yes, subjects with college education used HBPM more frequently than those with middle school education	NA	S^*∗*^C^*∗∗*^O^*∗∗*^ 5/10 Satisfactory

CSS = cross-sectional study, CVD = cardiovascular disease, Ref = reference number, *N* = number of hypertensive subject, NA = not available, HBPM = home blood pressure monitoring, HT = hypertension, BP = blood pressure, HL = health literacy, NOS = Newcastle-Ottawa score for cross-sectional studies, S = selection, C = comparability, O = outcome, OR = odds ratio.

**Table 3:** Summary of a selected randomized controlled trial.

Author	*N*	Level of education/Health literacy/Social factors	Intervention	Control	Outcomes	Any association between educational level and home BP monitoring?	Do the interventions result in better BP control?	Quality of evidence
Bachmann et al. [48], Switzerland, 2002	48	NA	Subjects received information about the storage capabilities of HBPM	Subjects did not receive information about the storage capabilities of HBPM	Manipulation of HBPM values for the first time. Accuracy and interpretation of HBPM may be increased by using devices with a memory	Y	NA	⊕⊕⊕ moderate
Binstock and Franklin [49], USA, 1988	120	NA	HBPM or a combination of techniques	Education alone, contract, or pill packs alone	SBP and DBP	NA	Y	⊕ low
Brenna et al. [50]. USA, 2010	485	94% ≥graduated high school	Telephonic nurse DM: educational materials, lifestyle, and diet counselling HBPM versus HBPM alone	Light support educational program	Increase proportion with BP < 120/80 mmHg, mean systolic BP, mean diastolic BP, and frequency of HBPM after the intervention	Y	Y	⊕⊕ low to moderate
DeJesus et al. [51], USA, 2009	54	NA	(1) Nurse educator conducted class + HBPM (2) Nurse educator class	Usual care	Only 20% achieved the target BP of 130/80 mmHg and there was no statistical difference in mean systolic and diastolic BP among the three groups	NA	N	⊕ low
Figar et al. [52], Argentina, 2006	60	Year of education	Compliance-based model includes HBPM	Patient empowerment model of education	Change in systolic BP by 24 h ABPM	NA	N	⊕⊕ low to moderate
Fung et al. [53], Hong Kong, 2003	240	NA	Individual education by research assistance of HBPM device operation Self-practice under supervision Checkpoints are correct	Usual care	No significant difference in BP changes between the two groups	Y	N	⊕⊕⊕⊕ moderate to high
Green et al. [54], USA, 2008	778	<12 years Some after high school 4-year college After 4-year college	HBPM and secure patient web services training + pharmacist care management delivered through web communications	Usual care	BP level	NA	Y	⊕⊕⊕⊕⊕ high
Haynes et al. [55], Canada, 1976	38	Steelworkers	Taught how to measure their own BP, chart their pill-taking, taught how to tailor pill-taking to their daily habits and rituals, FU by nonprofessionals	Usual care	Improvement in drugs compliance and BP	NA	Y	⊕⊕ low to moderate
Kauric-Klein and Artinian [56], USA, 2007	34	Year of education	HBPM	Usual care	Improvement in SBP but not DBP	NA	Y	⊕⊕⊕⊕ moderate to high
Kim et al. [57], USA, 2014	369	HT knowledge self-efficacy: HT belief scale; HT health literacy scale ≤ middle school graduate; high school graduate; ≥some college	2-hour weekly educational sessions × 6 on HBP management skill building, including health literacy training, followed by telephone counselling and HBPM for 12 months	Intervention delay	Intervention group showed improvement in mean SBP & DBP Improvement in health literacy in 12 and 18 months adherence to HT medication, self-efficacy, and HBP knowledge and less depression	NA	Y	⊕⊕⊕ moderate
Maciejewski et al. [58], USA, 2014	591	Completed <12 years of education	3 telephone-based interventions: nurse administered health behavior promotion Provider-administered medication adjustments based on HT treatment guideline Combination of both	Usual care	Patients randomized to the combined arm had greater improvement in the proportion of BP control during and after the 18-month trial	Y	Y	Unclear
Magid et al. [59], USA, 2011	338	High school education	Patient education including remote HBPM, reporting to an interactive voice response IVR phone system Pharmacists follow-up	Usual care	No difference in proportion of achieving BP goal at 6 months Reduction of mean SBP and DBP	NA	N	⊕⊕⊕ moderate
Morgado et al. [60], Portugal, 2011	197	Illiterate, elementary schooling, high schooling, university education	Quarter FU by a hospital pharmacist Provided patient education goal BP to achieve, medication education and recommendations to the physician regarding changes in drug therapy	No pharmacist care	Better medication adherence, significant lower SBP and DBP were observed in the intervention group	NA	Y	⊕⊕⊕⊕⊕ high
Nessman et al. [61], USA, 1980	52	Noncompliance patient	HBPM education patients select BP drugs emphasizing self-help informed program	Listened to audiotape on hypertension knowledge and management nurse adjusted the drug regimens	Lower DBP better pill counts better attendance	NA	Y	Unclear
Ogedegbe et al. [62], USA, 2014	1059	≤High school Some college Some graduate school	4 modules of interactive computerized patient education HBPM Monthly lifestyle counselling clinicians CME based training, HT case round, clinical audits of patient office BP readings	Patients and physicians received printed patient education material and hypertension treatment	Marginal significantly greater BP control in patients with moderate to good health literacy	NA	Y	⊕⊕⊕ moderate
Victor et al. [63], USA, 2011	1022	Black-owned barbershops </=high school college postgraduate	10 weeks of baseline BP screening offer BP checks with haircuts promote physician check-up Sex-specific peer-based health messaging	Received standard BP pamphlets	Improvement in hypertension control rate	NA	Y	⊕⊕⊕ moderate
Yi et al. [64], USA, 2015	900	Hispanic urban population Uninsured	Received a home blood pressure monitor and training on use	Usual care	No significant difference in BP changes	NA	N	⊕⊕ low to moderate
Yoo et al. [65], Korea, 2009	123	NA	Ubiquitous chronic disease care system using the cellular phone Internet for overweight patients	Usual care	Significant reduction of BP in the intervention group	NA	Y	⊕⊕⊕ moderate
Zillich et al. [66], USA, 2005	125	Pharmacists provided patient-specific education	Control group care + patient and physician educational program about hypertension treatment and monitoring	Provided with an HBPM device, instructed to measure BP > once daily for 1 month	More reduction of BP in the intervention group	NA	Y	⊕⊕ low to moderate

BP = blood pressure; DBP = diastolic blood pressure; FU = follow up; HBPM = home blood pressure monitoring; *N* = No; NA = not available/applicable; SBP = systolic blood pressure; Y = Yes.

## Abbreviations

BP: Blood pressure
C: Comparability
CVD: Cardiovascular disease
DBP: Diastolic blood pressure
EHS: European hypertension society
FU: Follow-up
HBPM: Home blood pressure monitoring
HL: Health literacy
N: No
NA: Not available/applicable
NOS: Newcastle-Ottawa Scale
O: Outcome
OR: Odds ratio
RCT: Randomized controlled trial
S: selection
SBP: Systolic blood pressure
SBPM: Self-blood pressure monitoring
Y: Yes.

## Supplementary Materials

Supplementary Materials Table 3: quality assessment of the selected interventional studies.
